# Existential Psychobiography: On Death and Dying Across the Life Span. Or How Extraordinary Individuals and Their Environments Deal With the End of Life

**DOI:** 10.5964/ejop.19961

**Published:** 2025-11-28

**Authors:** Claude-Hélène Mayer, Paul J. P. Fouché

**Affiliations:** 1 Department of Industrial Psychology and People Management, University of Johannesburg, Johannesburg, South Africa; 2 Department of Psychology, University of the Free State, Bloemfontein, South Africa; Stanford University, Stanford, USA

*For what* *is it to die*

*But to stand naked in the wind*

*And to melt into the sun?*

*And when the earth shall claim your limbs,*

*Then shall you truly dance.*

*– Kahlil Gibran*

## What is Death?

The question of how to define death and dying has become very complex during the past century based on new methodologies, new technologies and the discourses around what it means to be dead and to die (Veatch & Ross, 2016; Walter, 1994). It is an existential question (Belfrage, 1975). While there are various ways to explore this topic, death and dying are most commonly explored by focusing on physiological, social and psychological forms of death which can occur independently from each other and at different times during the lifespan (Butow, 2017).

According to Veatch and Ross (2016), there are mainly three discourses on concepts of physiological death. The first discourse views death as an irreversible loss of all brain functions; the second is based on the irreversible loss of circulatory function, and the third is associated with the loss of what came ambiguously to be called “higher-brain function”, and which is equated with consciousness. Based on these conceptualisations of death around physical functions of the human body, many of the discourses are anchored in the medical fields of study and biologistic definitions and terms. However, researchers from different disciplines such as philosophy, anthropology, psychology and sociology argue that death concerns not only “the irreversible loss of the integration of the organism as a whole” (Lizza, 2018) but also includes far-reaching discourses which go beyond physiological death and the biological functions of the body.

For example, Woodthorpe (2011, p. 100) does not refer to physiological death alone, declaring that “death is more than a sensitive subject – everyone is an ‘insider’ when it comes to death” owing to its “universal reach”. Social death and psychological death are two concepts that also play a role in death studies (Bryant, 2003; Hayslip & Hansson, 2003). Social death occurs when individuals withdraw from social interactions, while psychological death begins when the dying person accepts death and starts to withdraw psychologically from others and regresses into the self (Lazzara, 2020). Furthermore, research highlights that there is also spiritual death in addition to physiological, social and psychological death (Canadian Nurses Association, 2017).

Death, in any case, is an existential topic (Belfrage, 1975).

## The Development of Death Studies

Death studies have over the past decades seen different periods with various foci of academic research, reflection and practice (Borgstrom & Ellis, 2017; Howarth, 1996; Patra et al., 2025; Weenolsen, 1991). During the middle of the 20th century, death studies became relatively popular when several researchers such as Glaser and Strauss (1965) and Kübler-Ross (1969) created foundational works in the area of death and dying. This period of foundational death study research was followed in the 1990s by an increase in theoretical contributions and empirical research work by researchers including Mellor and Shilling (1993) and Young and Cullen (1996), in addition to subsequent edited death study collections such as that of Cann (2014). Death studies were taken from various disciplinary perspectives including sociology and psychology (Borgstrom & Ellis, 2017) and is part of existential psychology (Belfrage, 1975).

The 2010s witnessed several further assorted and innovative contributions to the field (e.g., Cann, 2014; Foster & Woodthorpe, 2016; Kellehear, 2014). An authoritative text titled *Handbook of Thanatology: The essential body of knowledge for the study of death, dying, and bereavement* provides a professional and practical research guide on dealing with human mortality and loss (Meagher & Balk, 2013). During the past decade, some prominent research which has come to fruition includes the work of Cano Molano (2024) titled *Death and grief: A complex thought point of view* and the applied research work undertaken by Canzona et al. (2023) titled *Piloting an interprofessional narrative-based interactive workshop for end-of-life conversations: Implications for learning and practice.*

In previous research, theories have been developed and applied to the study of death and dying through psychobiographical analysis (Mayer et al., 2021). These include developmental theories such as Piaget’s (1971) or Erikson’s (1980) theory, terror management theory (Becker, 1971, 1973; Greenberg et al., 1986), loss, bereavement and grief theories (Klass et al. 1996; Kübler-Ross & Kessler, 2014; Neimeyer, 2005; Neimeyer, 2016a, 2016b), care, spirituality, meaning and dying theories (e.g., Bonavita et al., 2018), euthanasia in different cultural and individual contexts (Gruman, 1973; Sabriseilabi, 2025), and *memento mori* (Pennington, 2001) or mortality awareness (Kuylen et al., 2024).

Recent studies reveal complex emotional, perceptual, spiritual, ethical, medical, social and cultural death journeys which have increasingly technological and legal implications (Cano Molano, 2024). However, individuals’ experiences of death and dying also need to be explored in more depth and from different methodological perspectives (Coenen & Meitzler, 2025; Woodthorpe, 2009) while taking various experiences and individual, sociocultural, economic, organisational, societal and global perspectives into consideration (Borgstrom & Ellis, 2017). Furthermore, different theories can be applied to describe, explore, analyse and better understand the perspectives on death and dying from within particular contexts, sociocultural environments and system dynamics (Ng et al., 2025; Pandey, 2025). If the social relationships are meaningful and constructive in the context of death and dying and across systemic networks, the dynamics and structures of individuals and groups can support a “good death” which is often presented as the ideal in death studies (Zarei et al., 2025).

Unpacking death and dying requires additional interdisciplinary understanding and a focus on three aspects. These are:

1. its social psychological, socio-psychological and socio-physiological processes.
2. its interactional dynamics and systems psychodynamics to understand the constructed relationship and structural processes around death and dying and resilience while experiencing loss, grief and bereavement.
3. a deeper understanding of agency and subjectivity regarding death and dying.

Kirmayer (2025, p. 771) emphasises that the interactional processes and the “cultural-ecosocial view on a subject matter constitute material and symbolic structures that provide cultural affordances, constraints, and challenges as well as resources for healing, recovery, and adaptation”. Therefore, it seems to be important to understand the person, death and dying from the perspective of their sociocultural, political and historical contexts.

Death is a natural process that is unavoidable. While death and dying are universal human experiences, they are deeply interwoven with sociocultural beliefs, values and practices which inform the ways in which individuals and societies deal with these experiences (Gutiérrez et al., 2020; Selman, 2024). Death and dying connect and affect individuals all over the world (Fenwick & Brayne, 2011; Kastenbaum & Moreman, 2018) and research on death, dying and bereavement is growing (Borgstrom & Ellis, 2017; Rolbiecki et al., 2025) while attitudes towards death and dying are shifting (Selman, 2024). Ideas about death and dying have an impact on how individuals conduct their lives and transition through the stages of life, particularly the final stages (Kastenbaum, 2004). According to research, individuals across the lifespan develop in the ways that they think about death, deal with it and approach it (Sala, 2025). Previous research has pointed out that death in an individual’s world often challenges their existing worldviews, especially when death is experienced as atypical, such as when it occurs due to suicide or the death of a child (Rolbiecki et al., 2025). Rolbiecki et al. (2025, p. 51) explain that even so-called “normal death” — which is defined as death in old age or death following prolonged illness — has often been under-researched in terms of sense-making and narratives around death and dying. Their study indicates that individuals aiming to make sense of a normal death in the family usually associate the death with “memorializing the whole person, reflecting on the caregiving experience, and emotional sense making” (Rolbiecki et al., 2025, p. 51).

Despite the accomplished and continually expanding collection of research, the study of death and dying is often still questioned. Previous research highlights that researchers in the field of death studies must frequently justify their research interest (Hockey, 2007). There are few significant publications devoted to in-depth, interpretative case studies of death and dying of historically eminent personalities. The field of psychobiography as a psycho-historical–interpretivist design and methodology (Fouché, 2025) could therefore be seen as a unique approach to the study of death and dying from a psychological perspective. Death studies might include the study of individuals’ cognitive approaches, emotions, rituals and religious practices in dealing with death and dying (Cano Molano, 2024), also from a psychobiographical viewpoint. Some studies do explore death and dying across the lifespan (Sala, 2025) and others explore grief, bereavement, mourning and death in extraordinary individuals (Stroebe & Schut, 1999, 2010, 2015) and how they manage to deal with death, dying and bereavement.

There is clearly a void in research on death and dying from a narrative and a lifespan research perspective (Bluck & Mroz, 2018). Having explored narrations of death, Bluck and Mroz (2018) argue that death is a part of life, a part of the life story of individuals and an act of solidarity between humans, since all humans have to die. Based on their literature review on death, there are two ways in which death can be considered as part of an individual’s life story. On the one hand, while individuals live, they experience endings of other lives. How people deal with loss, grief and bereavement contributes to their meaning-making in life and vice versa and is strongly connected to remembering the ones who have passed on and the memories about them (Bluck et al., 2010). On the other hand, each individual will die and every person will experience their own death. While individuals are aware of the fact that life will one day end, the way in which they make sense of death, how they understand it and how they feel about it, influences how they conduct their lives (Bluck & Mroz, 2018).

This special issue deals with death and dying in the lives of extraordinary individuals. In the following, the editors define the purpose, aim and contribution of this special issue. They explore definitions of death and dying, discuss death studies across the lifespan and in psychobiography, provide a perspective on suicide and briefly present the contributions to this edited volume.

## Death Studies Across the Lifespan and in Psychobiography

Psychobiography is on the rise in the 21^st^ century (Anderson, 2025; Elms, 1994; Mayer & Kovary, 2019; Ponterotto, 2025; Schultz, 2005). In psychobiography, researchers primarily use psychological theories, psychobiographical and psychohistorical approaches to explore, describe, analyse, discuss and interpret the lives of extraordinary individuals (Mayer et al., 2023). Recently, psychobiography researchers have called for the increased use of interdisciplinary theoretical approaches and new theoretical foci in psychobiography and lifespan narratives (Mayer et al., 2021, 2023).

Prior research has investigated how individuals experience death and dying from a lifetime perspective (Clark, 1993; de Vries et al., 1993; Hockey, 1990, 2007; Howarth, 1993; Kendall, 2007; Kendall et al., 2007; McIntosh, 1998; Murray et al., 2007; Visser, 2017; Webb et al., 2007). de Vries et al. (1993), for example, have researched how individuals write about and discuss death and dying during a lifetime. On the one hand, their research reveals that individuals were more concerned with and focused on death than they were on dying. On the other hand, middle-aged writers were more concerned with dying than were any of the other age groups. Further, individuals spoke more about the death and dying of others than of their own death and dying. However, when they did speak about themselves, they did so in more detailed ways than when talking about others. This research study suggests that it might be easier to discuss death and dying when exploring the topics on behalf of others rather than for oneself.

Psychobiography explores the life of and specific events during the lifespan of extraordinary individuals (Elms, 1994). Aspects of death and dying, as part of every individual’s life, are therefore often part of psychobiography. However, psychobiographers have seldom focused on death and dying in the lives of extraordinary individuals. This could be due to the possibility that researchers investigating a certain topic might experience the blurring of boundaries between the researcher and the self (Woodthorpe, 2011) and might through resonance with own experiences of death, dying, loss or grief be stimulated to reflect upon their own thoughts, emotions and feelings more than usual throughout their lives. Researchers have pointed out that those researching death and end-of-life issues could experience higher levels of general anxiety about mortality, but also about the uncertainty and fragility of life, about illness and possibilities of terminal illness (Mckenzie et al., 2017).

Psychobiography, as a specific research methodology, often explores life developments in extraordinary individuals across the lifetime or specific outstanding life events (Mayer & Kovary, 2019; Wegner, 2020). Accordingly, psychobiographers deal in particular with biographical and narrative accounts (Mayer, 2023). Personal narratives in the study of death and dying have often been assumed to provide primary sources of essential meaning (Kavas, 2018) and different forms of narrative can support individuals to manage dying and death experiences by processing emotions, sharing experiences and reflecting upon the experiences of others (Canzona et al., 2023). Ellis (2003), for example, has written about death experiences from autoethnographical perspectives, examining family rituals around death, feeling, connections to the loss and respect for the passed-on family members, thereby starting to explore the researcher’s own ideas with regard to her own death. Ellis (1993) highlights that the reading of personal narratives and stories of death can help to reposition the reader vis-à-vis the author of the text with regard to experiencing the feelings and thoughts of the writer through the reader. The author highlights that by reading about the lived experience, the reader can “acknowledge and give a voice to their own emotional experiences” (Ellis, 1993, p. 711).

Psychobiography and the in-depth analysis of individuals’ lives — to a certain extent as autobiographies — can provide rigorous knowledge and emotional understanding of how individuals deal with death and dying throughout their lifetime, at the end of their lives and how they create meaning in their sociocultural contexts (Mayer, 2021; Mayer et al., 2021). At the same time, they can further lead to reliving and recreating the described emotions of the subject in the mind of the writer and reader.

Death study researchers observe that cultural and diverse dying and death experiences are often absent from or seldom reflected in research on death and dying (Kellehear, 2009). Psychobiographies may open the door to exploring death and dying from increasingly diverse sociocultural, gendered and age-related perspectives and contribute to innovative and original knowledge on death and dying across the lifespan in specific sociocultural and historical contexts. This form of study can also open the doors to an emotional experience of mortality through reflection and experience of death-related life events. Psychobiography thereby becomes a tool of reflection and might even, in selected instances, provide new and original insights into how to overcome grief regarding death and dying, how to create increasing meaning in life in the face of human mortality, or how to become aware of risk factors regarding specific forms of death such as suicide.

Many psychobiographies explore the lives of extraordinary individuals holistically, across the lifespan and in chronological order (Ponterotto, 2015); however, they seldom focus on the life sequence of death and dying. So far, only very few psychobiographies exist that focus on death and dying, such as Yeh and Trang’s (2022) psychobiographical work on the Swedish artist Avicii which explores suicide and suicidal ideation in the context of emotional and mental distress, dislocations of emotions, and complex interpersonal relationships. Elovitz’s (2017) work also researches the death drive of selected individuals through psychobiography. Recently, Misra and Srivastava (2021) explored suicide among extraordinary individuals and related it to happiness and well-being, while investigating reasons for the high suicide numbers. It seems that suicide, as an extraordinary way of dying (Misra & Srivastava, 2021; Yeh & Trang, 2022), has caught the attention of selected psychobiographers, although the process of death and dying in general has been rather neglected in psychobiographical research.

To summarise, it is argued here that psychobiography as a narration of the life and death of extraordinary individuals, based on psychological theories, can increase reflection, discourses and new perspectives for psychobiographers, interdisciplinary scientists and readers of the psychobiographical accounts. This is especially the case since psychobiographers have recently called for the practical applications of the “lessons learned” from psychobiography of extraordinary individuals to contemporary life situations (Mayer & Fouché, 2021).

## Suicide as a Specific Form of Death and Dying

In this special issue, three of the six articles deal with a specific form of death and dying, namely suicide. According to the National Institute of Mental Health (2025), suicide is defined as “death caused by self-directed injurious behavior with intent to die as a result of the behavior”. Suicide occurs worldwide and is one of the leading causes of death in the United States of America (National Institute of Mental Health, 2025). The World Health Organization (WHO, 2025) points out that the reasons for suicide are multi-faceted, being influenced by social, cultural biological, psychological and environmental factors which can occur at any point across the lifetime.

Suicides can be connected to the political and sociocultural context and the experience of social justice or social injustice (Button, 2016). Furthermore, suicide always needs to be understood in the context of the individuals and their sociocultural, historical and political background (Staples & Widger, 2012).

Suicide is often associated with psychological pain (Baryshnikov & Isometsä, 2022) or mental pain (Meerwijk & Weiss, 2014) and psychache (Shneidman, 1993, 1998). There is a strong link between suicide and mental disorders (WHO, 2025). Often there are very complex factors associated with suicide. Risk factors for suicidal behaviour include, for example, anxiety, depression, hopelessness and psychological pain, and the differentiation of these emotional and cognitive concepts is not always easy to ascertain (Baryshnikov & Isometsä, 2022). In their edited work, Weaver and Wright (2009) provide a helpful insight into suicide in different sociocultural contexts, countries, at different historical times and in connection with different risk factors. They also offer different perspectives on and interpretations of suicide and how it can be understood in the context of war, homicide, medico-legal disputes, and racialised and gendered contexts. Previous work shows that the complexity of suicide requires in-depth research and interpretations. Researchers may use multiple methodological approaches and theories to increasingly understand suicide in its depth.

Suicide has been researched in prior psychobiographical studies (Citlak, 2024; Hamilton, 2016; Kitching, 2017; Kramer, 2002; Smith, 2014; Zanalda, 1968), creating suicide awareness towards a comprehensive and holistic approach to suicide prevention and defining protective factors to influence suicidal behaviours. Similarly, another psychobiography deals with the life of James Baldwin, an African American author who did not die from suicide, but who carried out several suicide attempts and was consumed by suicide ideation and thoughts of committing suicide throughout his life (Reynolds et al., 2023). It has been noted in previous research on artistic creativity that writers and poets tend to commit suicide more often than artists do, while musicians have the lowest suicide rates (Preti et al., 2001; Preti & Miotto, 1999). Hamilton (2016) published an article about the suicides of four famous individuals — Socrates, Cleopatra, Kurt Cobain, and Hemingway — and highlighted that often suicide is highly complex and needs to be understood in the context of mental health, various environmental risk factors, goal-setting and achievement, as well as in terms of heroism and martyrdom, fear of old age and the experience of a potential loss of success and fame when the level of fame and success are significant.

The following six articles address the topic of death and dying. Three of them explore death and dying when manifested in suicide. All articles use different theoretical perspectives. However, they all use psychobiography as their research methodology to describe, explore, analyse and interpret the topic. The next section briefly introduces each of the articles in this special issue.

## The Purpose, Aim and Contribution of This Special Issue

The purpose of this special issue is to bring death studies and psychobiography together to explore the lives of extraordinary individuals with regard to the topic of death. The idea of compiling a special issue on death and dying in psychobiography arose for the first editor while dealing with loss, grief and bereavement due to the experience of terminal illness, suffering and loss of close family members and friends. The following questions arose during the time of suffering: “How do individuals die?” and “How do the individuals who remain carry on with their lives and deal with death and dying and endings of life around them?”. Further questions that came to mind were: “Which narrations do individuals construct around their own death, and which around the death of others?”, “Which stories and narrations do individuals actually create during their lifetime around their approaching, future death?” and “When do individuals commit suicide and if they do, how do the circumstances during their lifetime lead to the suicide?”. Based on these reflective questions, I invited my co-editor Professor Paul J. P. Fouché to create a special issue on death and dying together with me, particularly focusing on death and dying in extraordinary individuals through psychobiography (Fouché & van Niekerk, 2010; Mayer & Kovary, 2019; Schultz, 2005).

The editors aim with this special issue to explore death and dying in extraordinary individuals based on the perspective that extraordinary individuals often leave a meaningful legacy behind them. This legacy might assist in creating a sense of meaning and a sense of purpose in life, thereby helping when being confronted with one’s own mortality (Holloway, 2017). Readers of psychobiographies on death and dying might be stimulated to reflect upon their own finitude, their own sense of purpose and their own sense of meaning and might develop a greater acceptance of the process of the death and dying of their own life story.

Death is viewed here as a complex and holistic process focusing not only on physical functions, but rather on the causes of death and dying, the experiences of it in selected extraordinary individuals, and the effects of their death and dying within their living environments. In this way, death is not understood as a single life event but is rather viewed as a continuous development process across the lifespan (Weenolsen, 1991). It is further assumed that death and dying are transcendental aspects and dynamics of life which affect life narrations but also influence the identity and self-constructions of individuals, thereby creating and recreating their lives in the face of death.

Summarising, the contributions of this special issue are multifold:

- It integrates death and dying theories and death studies with psychobiography.
- It explores and expands on theories of death and dying based on description, analysis and reflection on death and dying within the life and times of selected extraordinary individuals.
- It aims to encourage reflection in the reader on death and dying of individuals within their systemic contexts.
- It aims to construct a deeper understanding of the interconnection of life and death, with death as a part of life and within the environment of extraordinary individuals from a perspective of death and dying.
- It uses different research methodologies in the psychobiographical paradigm to explore death and dying at specific points across the lifespan of individuals.

The special issue was stimulated by the questions presented above. The articles in this special issue touch, in part, on the questions and respond to them. This issue could be viewed as a beginning point to explore death and dying in a psychobiographical context and might be viewed as pioneering work in creating interdisciplinary and intercultural psychobiographies and psycho-historical work in the context of the finite life.

## A Brief Description of the Articles in This Special Issue

This special issue on “Death and Dying in Psychobiography” aims at connecting psychobiography, interdisciplinary theories and practical applications for managing in-depth personal views regarding, and experiences of, death and dying. Contributions in this issue connect to articles that were previously published in the journal which relate to psychobiography (DeArmond, 2013). The articles explore, describe, analyse and interpret final life stages, dying and death in extraordinary individuals, including specific incidents such as terminal illness, fatal accidents, catastrophe, suicide and bereavement. Further on, they contribute to investigating how sociocultural and personal beliefs, emotions and behaviours influence how extraordinary individuals deal with death and dying and how their death and dying relates to the relationships in their lives, their loneliness and isolation.

The special issue begins with an article by **Paul J. P. Fouché** on “*Heroic death: A melancholic existentialist psychobiography of Jacques de Molay*”. In this psychobiography, the author aims to reconstruct the life and death of Jacques de Molay (1243–1314), who was the Grand Master of the Knights Templar. The author focuses on de Molay’s heroic struggle as a mitigating psychological mechanism against the terror of mortality. De Molay was part of an order of knighthood founded during the Crusades in the late 11^th^ century and dedicated his life to the mission of protecting Christian pilgrims and defending the Holy Land. He was confronted by betrayal, arrest, torture, confessions of heresy under duress and public execution. According to the researcher, de Molay’s life offers an impactful illustration of the melancholic existentialist theory of Ernest Becker, who posited that mortality creates profound existential anxiety. Salient themes and events were interpreted via Becker’s melancholic existential theory, with particular emphasis on heroism. The torture he endured and his refusal to betray the Templars’ ideals exemplify the theory of Becker’s heroic struggle against mortality. Instead of capitulating to terror and falsehood, de Molay faced his death maturely, thereby achieving existential authenticity and symbolic immortality.

The second article in this special issue is titled “*Death and dying in the life and creative works of Camille Rosalie Claudel”*, written by **Claude-Hélène Mayer.** This article explores the themes of death and dying in the life and creative work of the French sculptress Camille Rosalie Claudel (1864–1943). Claudel was a talented artist and sculptress who learned from and worked with Alfred Boucher and Auguste Rodin. In her psychobiography, the author uses four theoretical approaches to explore death and dying in Claudel’s life and work: existential psychology theories on death and dying, symbolic death theory, Lacanian death theory and Jung’s perspectives on death, dying and *ars moriendi.* Findings show that through her art, Claudel expressed her inner life and many important aspects of her life’s journey, her search for meaning, her artistic talent and in-depth philosophical interest, but also her views on life and death. Throughout her lifetime, she experienced different forms of death and dying which the author discusses in this psychobiographical work on Claudel.

The third article, contributed by **Carla Nel**, is “*Psychobiographical reflections on Marilyn Yalom’s experience of death and dying”.* Marilyn Yalom (1938–2019) was a distinguished feminist scholar and cultural historian. She documented her intimate reflections on dying in several written accounts, partly co-authored with her husband Irvin Yalom who is a highly renowned existential psychotherapist and author. Nel seeks to examine Marilyn Yalom’s thoughts and experiences concerning dying by focusing primarily on one written source, her diary entries, while living with and dying from a terminal illness. The author focuses on describing Yalom’s reflections through an existential psychology lens to construct a theoretically informed overview of her experiences. In the focus are her reflections on facing death, the process of dying and her insights regarding her transition into death. Discourses are centred on how to fight against despair and how to live meaningfully until the very end. This study primarily provides an in-depth existentially informed analysis of Yalom’s final reflections on her death and uncover additional themes of her experiences of dying to fully explore her insights on aspects such as physical impairment, relationships and separations, and physician-assisted suicide.

The following article on *“Lawrence Kohlberg’s suicide: An analysis of his final life stage”*, written by **Vikki Botes** and **Roelf van Niekerk**, also deals with the topic of suicide. Lawrence Kohlberg (1927–1987) was a pioneer in moral development studies. He fought in the Second World War and helped smuggle refugees from Europe into Palestine, dedicating his life to preventing similar horrors from occurring in future. Kohlberg became famous in 1958 after he published pioneering research on moral development. However, he died tragically when he committed suicide in 1987. The authors use the theoretical framework of Bronfenbrenner’s bioecological theory of human development to explore his life. They further incorporate Bronfenbrenner’s proximal-person-context-time model to understand the life dynamics within systemic contexts. The article focuses on specific events during the last stages of Kohlberg’s life from 1968 to 1987 and provides a deep insight into this phase of the end of his life.

The next article, written by **Amadeusz Citlak,** is called *“Socrates’ voluntary death – An essential voice against the pathologisation of suicide”*. The article deals with the death of Socrates, a prominent philosopher living in the 5^th^ century BCE, and uses it as a starting point for a discourse on suicide. The author uses a single-case analysis with an interpretive-descriptive approach as a theoretical starting point to highlight how an individual and their environment interact when dealing with the problem of death. On analysis, the concept of honour shame is identified as an important dimension both in ancient Greek culture and in the context of the universal concept of social status. The article provides a perspective on Socrates and thereby allows the reader to receive an alternate perspective on suicide and the right to suicide.

In the final article, **Virag Rab** writes about “*Death and Melody: A psychobiography of Rezső Seress, composer of Gloomy Sunday*”. Rezső Seress (1899–1968) was a Hungarian composer and pianist who composed the song “Gloomy Sunday”. The song became an international success and, in the collective memory, a symbol of death and suicide. The life of Seress, however, ended tragically: he made two suicide attempts, the second of which caused his death. The author responds to the research question of how one can interpret Seress’s act and places it in a cultural, linguistic, social and historical context. At the same time, this psychobiography uses an integrated theoretical framework by applying Kézdi’s theory of the negative code, Wallerstein’s world systems theory, and McAdams’s theory of narrative identity (McAdams, 1993). This study proposes a narrative type derived from historical, cultural, and semiperipheral contexts, called the tragic semiperipheral self. Furthermore, Kézdi shows how Seress’s suicide can be read culturally. Wallerstein’s theory reveals how Hungary’s semiperipheral structural position and instability influence meaning-making. The concept of the tragic semiperipheral self helps the reader to understand how Seress constructed his personal narrative.

## Conclusions

Unpacking death and dying in psychobiography is the core theme of this special issue, which aims to fulfil the purpose of bringing death studies and psychobiography together. In focus are the lives and deaths of selected extraordinary individuals. The authors examine predominantly historical subjects whose lives present excellent examples to describe, explore, analyse, and interpret death and dying in more depth. The authors respond to a variety of research questions, thereby aiming to contribute to the existing literature and theories concerning death and dying, with particular focus on suicide.

This special issue is a vivid account of explorations of experienced life and death, lived life and death, and narrated life and death. It showcases the stories that are created from etic perspectives, and the authors’ minds, to understand the life and death of extraordinary individuals in their sociocultural environments and historic and contemporary contexts.

It can be concluded that death and dying, like life, are very much connected to the creating of meaning, sense-making and purpose in life. Every life, one day, comes to an end. This special issue is intended to create awareness, interest and deep reflection and support individuals to engage with the finitude of life. In the end, it aims to ask the questions: How would you, the reader, live your life differently, if you were aware in every second of your life, that it will end? How would you care, in every second, about others? Which values would you follow; which actions would you take?

This special issue not only integrates death studies and psychobiography. It also aims to create reflections on the life and death of the individuals in their systemic environments and construct a deeper understanding of the interconnections and systemic dynamics in our lives when it comes to how we live, which meanings we apply to our lives and how we die. The articles presented in this special issue might increase awareness of the finitude of life and make a difference in the choices readers make after reading the articles. Through the application of the research methodology of psychobiographical work, this issue provides examples of death and shows that dying can happen in many different ways and that it is very much connected to us as individuals and to the ways in which we lead our lives within our created contexts.

## The Way Forward: Existential Psychobiography

The future of the integration of death studies and psychobiography is bright. Future research can further explore the individual perceptions of death and dying in individual extraordinary lives, thereby responding to questions such as: How can death studies be developed through in-depth psychobiographical research? How do individuals die, and which influences in their environments influence their death? What leads to suicide in the lives of extraordinary individuals? How do individuals deal with the suicide of others when experienced during a lifetime?

Various theories have already been used in the sciences to explore death and dying, such as Freud’s thanatology, Erikson’s psychosocial stages, Kubler-Ross’s five stages of grief and even attachment theory. However, several of these theories have not yet been applied in psychobiographical accounts across times and contexts. These theories can further be explored in more depth and refined through psychobiography. Psychobiographical discourses will also need to explore the ethical considerations when dealing with sensitive issues such as confidentiality and sensitivity of the topics of death and dying and suicide. Because death, trauma, grief and bereavement might be activated in doing and reading research on death and dying, the topic needs to be treated with care, ethical consideration and respectful and benevolent approaches.

Potential future areas for psychobiographical death studies may involve existential reflection and the awareness of death and dying in the lives of extraordinary individuals, as well as their near-death experiences or the experience of terminal illness. How do these themes affect the mind at in-depth intra-individual levels?

More research will probably be undertaken in psychobiography and death anxiety, exploring how the fear of death influences decision-making in life, major life choices and the mindset throughout life. It is also likely that positive psychology approaches may be combined with death studies while using psychobiographical methods. This could lead other authors to explore positive psychology theories to be applied to the lives of extraordinary individuals to further examine ways to overcome grief, bereavement, death anxiety and create post-traumatic growth and transformational spaces in life. Another interesting future research direction would be to focus psychobiographical studies on the review of life, on the meaning of life and its purpose, and on the differences between death, dying and suicide in extraordinary individuals in comparison to less extraordinary individuals. Do extraordinary individuals die differently to others?

In terms of the future design of psychobiographies, psychobiographers might combine the design of exploring an entire life with focusing only on specific life events. Psychobiographies might become more integrated with other theories and subdisciplines such as neuropsychology and death studies, or even epigenetics, to investigate, for example, brain processes, death-related thoughts, grief and trauma and their consequences in the life histories of specific individuals. This will further require new combinations of psychobiographical research with thanatological theories which might include the exploration of death rituals in selected lives, the impact of sociocultural norms and family influences on grief, as well as the psycho-philosophical reflections on afterlife from the perspective of extraordinary individuals.

Finally, new research will need to explore how reading psychobiographies on death and dying affect the readers of those psychobiographies. Thereby, the writers and readers of psychobiographies on death and dying might ask themselves questions, such as: How does the psychobiographical account create a connection to the reader and what does the reader gain from it? How are psychobiographical and autoethnographic narrations intertwined, especially when it comes to meaning-making, death and dying?

This special issue creates a new level of psychobiography, here referred to as “**existential psychobiography**” since it aims to understand not only the lives of extraordinary individuals, but also the existential core of life: its finitude and end and therefore the existential essence of human existence.

## References

[r1] Anderson, J. W. (2025). *Psychobiography. In search of the inner life*. Oxford University Press.

[r2] Baryshnikov, I., & Isometsä, E. (2022). Psychological pain and suicidal behavior: A review. Frontiers in Psychiatry, 13, 981353. 10.3389/fpsyt.2022.981353 36203837 PMC9531162

[r3] Becker, E. (1971). *The birth and death of meaning* (2^nd^ ed.). Free Press.

[r4] Becker, E. (1973). *The denial of death* (1^st^ ed.). Free Press.

[r5] Belfrage, L. (1975). *The role of the concept of death in existential psychology: From Kierkegaard to Binswanger*, Paper 2124. Masters Theses & Specialist Projects: TopSCHOLAR. https://digitalcommons.wku.edu/theses/2124

[r6] Bluck, S., Alea, N., & Demiray, B. (2010). You get what you need: The psychosocial functions of remembering. In J. Mace, (Ed.), *The act of remembering: Toward an understanding of how we recall the past*. Wiley-Blackwell. 10.1002/9781444328202.ch12

[r7] Bluck, S., & Mroz, E. L. (2018). The end: Death as part of the life story. International Journal of Reminiscence and Life Review, 5(1), 6–14.

[r8] Bonavita, A., Yakushko, O., Morgan Consoli, M. L., Jacobsen, S., & Mancuso, L. L. (2018). Receiving spiritual care: Experiences of dying and grieving individuals. Omega, 76(4), 373–394. 10.1177/0030222817693142 29284323

[r9] Borgstrom, E., & Ellis, J. (2017). Introduction: Researching death, dying, and bereavement. Mortality, 22(2), 93–104. 10.1080/13576275.2017.1291600

[r10] Bryant, C. D. (Ed.). (2003). *Handbook of death and dying.* SAGE Publications.

[r11] Button, M. E. (2016). Suicide and social justice: Toward a political approach to suicide. Political Research Quarterly, 69(2), 270–280. 10.1177/1065912916636689

[r12] Butow, P. (2017). Psychology and end of life. Australian Psychologist, 52(5), 331–334. 10.1111/ap.12306

[r13] Canadian Nurses Association. (2017). *Ethics in practice: A guide for ethical decision making*. Canadian Nurses Association.

[r14] Cann, C. K. (2014) *Virtual afterlives: Grieving the dead in the twenty-first century.* University Press of Kentucky.

[r15] Cano Molano, L. M. (2024). Death and grief: A complex thought point of view. In G. Bollig & E. Zelko (Eds.), *Palliative care — Current practice and future perspectives.* IntechOpen. 10.5772/intechopen.1003065

[r16] Canzona, M. R., Love, D., Barrett, R., Henley, J., Bridges, S., Koontz, A., & Nelson, S. (2023). Piloting an interprofessional narrative-based interactive workshop for end-of-life conversations: Implications for learning and practice. Omega, 86(3), 862–888. 10.1177/0030222821993633 33557720

[r17] Citlak, A. (2024). Desire for a sense of power and religious suicide in psychobiographical research: Combining personality and sociocultural theories. International Review of Psychiatry, 36(1–2), 165–179. 10.1080/09540261.2023.2260462 38557337

[r18] Clark, D. (Ed.). (1993). *The sociology of death: Theory, culture, practice*. Blackwell Publishers/Sociological Review.

[r19] Coenen, E., & Meitzler, M. (2025). Exploring death, dying, and bereavement: Characteristics and challenges of a sensitive field of research. In P. Liamputtong (Ed.), *Handbook of sensitive research in the social sciences* (pp. 77–92). Edgar Elgar Publishing.

[r20] DeArmond, I. M. (2013). The psychological experience of hospice workers during encounters with death. Omega, 66(4), 281–299. 10.2190/OM.66.4.a 23785981

[r21] de Vries, B., Bluck, S., & Birren, J. E. (1993). The understanding of death and dying in a life-span perspective. Gerontologist, 33(3), 366–372. 10.1093/geront/33.3.366 8325524

[r22] Ellis, C. (1993). There are survivors: Telling a story of sudden death. Sociological Quarterly, 34(4), 711–730. 10.1111/j.1533-8525.1993.tb00114.x

[r23] Ellis, C. (2003). Grave tending: With Mom at the cemetery. Forum Qualitative Sozialforschung / Forum: Qualitative Social Research, 4(2). 10.17169/fqs-4.2.701

[r24] Elms, A. C. (1994). *Uncovering lives. The uneasy alliance of biography and psychology*. Oxford University Press.

[r25] Elovitz, P. H. (2017). Ambivalence about Freud’s death instinct and diverse views of suicide. Clio’s Psyche, 23(3), 277–281. 10.70763/dccab9bd9e0b69c49302025efdf19702

[r26] Erikson, E. H. (1980). *Identity and the life cycle.* W. W. Norton.

[r27] Fenwick, P., & Brayne, S. (2011). End-of-life experiences: Reaching out for compassion, communication, and connection — Meaning of deathbed visions and coincidences. American Journal of Hospice & Palliative Care, 28(1), 7–15. 10.1177/1049909110374301 20801918

[r28] Foster, L., & Woodthorpe, K. (Eds.). (2016). *Death and social policy in challenging times.* Palgrave Macmillan.

[r29] Fouché, P., & van Niekerk, R. (2010). Academic psychobiography in South Africa: Past, present and future. South African Journal of Psychology = Suid-Afrikaanse Tydskrif vir Sielkunde, 40(4), 495–507. 10.1177/008124631004000410

[r30] Fouché, P. J. P. (2025). The legacy of Pierre Jaquet-Droz and his humanoid automata: A Jungian archetypal-interpretivist psychobiography of the writer, draughtsman and musician. International Review of Psychiatry, 37(5), 416–426. 10.1080/09540261.2025.2466504 40970892

[r31] Glaser, B. G., & Strauss, A. L. (1965). *Awareness of dying*. Aldine Transaction.

[r32] Greenberg, J., Pyszczynski, T., & Solomon, S. (1986). The causes and consequences of a need for self-esteem: A terror management theory. In R. F. Baumeister (Ed.), *Public self and private self* (pp. 189–212). Springer-Verlag.

[r33] Gruman, G. J. (1973). An historical introduction to ideas about voluntary euthanasia: With a bibliographic survey and guide for interdisciplinary studies. Omega, 4(2), 87–138. 10.2190/A7WG-A6EQ-0XFP-9T4J 11664247

[r34] Gutiérrez, I. T., Menendez, D., Jiang, M. J., Hernandez, I. G., Miller, P., & Rosengren, K. S. (2020). Embracing death: Mexican parent and child perspectives on death. Child Development, 91(2), e491–e511. 10.1111/cdev.13263 31140591 PMC6883122

[r35] Hamilton, A. (2016). Four famous suicides in history and lessons learned: A narrative review. Mental Health & Prevention, 4(3–4), 138–145. 10.1016/j.mhp.2016.08.001

[r36] Hayslip, B., & Hansson, R. O. (2003). Death awareness and adjustment across the life span. In C. D. Bryant, P. M. Bryant, C. K. Edgley, M. R. Leming, D. L. Peck, K. L. Sandstrom & W. F. Rogers (Eds.). *Handbook of death and dying* (pp. 437-447). SAGE.

[r37] Hockey, J. (1990). *Experiences of death. An anthropological account*. Edinburgh University Press.

[r38] Hockey, J. (2007). Closing in on death? Reflections on research and researchers in the field of death and dying. Health Sociology Review : The Journal of the Health Section of the Australian Sociological Association, 16(5), 436–446. 10.5172/hesr.2007.16.5.436

[r39] Holloway, K. (2017). Leaving a legacy: Creating a narrative for a meaningful end of life. Death Studies, 41(5), 295–302.

[r40] Howarth, G. (1993). Investigating deathwork: A personal account. In D. Clark (Ed.), *Sociology of death. Theory, culture, practice* (pp. 221–237). Blackwell Publishing/Sociological Review.

[r41] Howarth, G. (1996). *Last rites: The work of the modern funeral director*. Baywood.

[r42] Kastenbaum, R. (2004). *On our way: The final passage through life and death. *University of California Press.

[r43] Kastenbaum, R., & Moreman, C. (2018). *Death, society, and human experience* (11^th^ ed.). Routledge.

[r44] Kavas, M. V. (2018). How to increase the quality of a suffering experience: Lessons derived from the diary narratives of a dying adolescent girl. Omega, 76(3), 256–295. 10.1177/0030222817694667 29279037

[r45] Kellehear, A. (2009). What the social and behavioural studies say about dying. In A. Kellehear (Ed.), *The study of dying: From autonomy to transformation* (pp. 1–26). Cambridge University Press.

[r46] Kellehear, A. (2014). *The inner life of the dying person*. Columbia University Press.

[r47] Kendall, S. (2007). Witnessing tragedy: Nurses’ perceptions of caring for patients with cancer. International Journal of Nursing Practice, 13(2), 111–120. 10.1111/j.1440-172X.2007.00615.x 17394519

[r48] Kendall, M., Harris, F., Boyd, K., Sheikh, A., Murray, S. A., Brown, D., Mallinson, I., Kearney, N., & Worth, A. (2007). Key challenges and ways forward in researching the ‘good death’: Qualitative in-depth interview and focus group study. BMJ (Clinical Research Ed.), 334(7592), 521. 10.1136/bmj.39097.582639.55 17329313 PMC1819552

[r49] Kirmayer, L. J. (2025). The place of the social in psychiatry: From structural determinants to the ecology of mind. Social Psychiatry and Psychiatric Epidemiology, 60, 771–783. 10.1007/s00127-024-02772-5 39340545

[r50] Kitching, P. H. (2017). *Understanding suicide: A psychobiographical study of Ian Kevin Curtis*. Doctoral thesis. NMU, Port Elizabeth. http://vital.seals.ac.za:8080/vital/access/manager/Repository/vital:28583?site_name=GlobalView

[r51] Klass, D., Silverman, P. R., & Nickman, S. L. (Eds.). (1996). *Continuing bonds. New understandings of grief.* Taylor & Francis.

[r52] Kramer, D. A. (2002). A psychobiographical analysis of faith, hope, and despair. Journal of Adult Development, 9(2), 117–126. 10.1023/A:1015785313076

[r53] Kuylen, M., Han, S., Harris, L., Huys, Q., Monsó, S., Pitman, A., Fleming, S. M., & David, A. S. (2024). Mortality awareness: New directions. Omega, 90(1), 143–157. 10.1177/00302228221100640 35531947 PMC11437703

[r54] Kübler-Ross, E. (1969). *On Death and dying* (1^st^ ed.). Routledge. 10.4324/9780203010495

[r55] Kübler-Ross, E., & Kessler, D. (2014). *On grief and grieving: Findings the meaning of grief through the five stages of loss.* Scribner.

[r56] Lazzara, J. (2020). *Lifespan development: Death and grief*. Maricopa Community Colleges. https://open.maricopa.edu/devpsych/chapter/chapter-12-death-and-dying/

[r57] Lizza, J. P. (2018). Defining death: Beyond biology. Diametros: A Journal of Philosophy, 55, 1–19. 10.13153/diam.1172

[r58] Mayer, C.-H. (2021). Albert Camus — A psychobiographical approach in times of Covid-19. Frontiers in Psychology, 12, 644579. 10.3389/fpsyg.2021.644579 33796053 PMC8007927

[r59] Mayer, C.-H. (2023). Psychobiographical trends: Untold stories and international voices in the context of social change. Journal of Personality, 91(1), 262–265. 10.1111/jopy.12771 36031389 PMC10108238

[r60] Mayer, C.-H., & Fouché, P. J. P. (2021). Lessons learnt from Baruch Spinoza: Shame and faith development in the light of challenges in contemporary society. In C.-H. Mayer, E. Vanderheiden & P. T. Wong. (2021). *Shame 4.0: Investigating an emotion in digital worlds and the Fourth Industrial Revolution* (p. 247–274). Springer. 10.1007/978-3-030-59527-2_13

[r61] Mayer, C.-H., & Kovary, Z. (2019*). **New trends in psychobiography*. Springer.

[r62] Mayer, C.-H., Krasovska, N., & Fouché, J. P. (2021). The meaning of life and death in the eyes of Frankl: Archetypal and terror management perspectives. Europe’s Journal of Psychology, 17(3), 164–175. 10.5964/ejop.4689 35136437 PMC8763221

[r63] Mayer, C.-H., van Niekerk, R., Fouché, P. J., & Ponterotto, J. (2023). *Beyond WEIRD: Psychobiography in times of transcultural and transdisciplinary perspectives.* Springer.

[r64] McAdams, D. P. (1993). *The stories we live by: Personal myths and the making of the self.* William Morrow & Co.

[r65] McIntosh, J. L. (1998). Death and dying across the life span. In T. L. Whitman, T. Merluzzi & R. D. White. (Eds.), *Life-span perspectives on health and illness*. Psychology Press.

[r66] Mckenzie, S. K., Li, C., Jenkin, G., & Collings, S. (2017). Ethical considerations in sensitive suicide research reliant on non-clinical researchers. Research Ethics, 13(3–4), 173–183. 10.1177/1747016116649996

[r67] Meagher, D. K., & Balk, D. E. (Eds.). (2013). *Handbook of thanatology: The essential body of knowledge for the study of death, dying, and bereavement* (2^nd^ ed.). Routledge. 10.4324/9780203767306

[r68] Mellor, P. A., & Shilling, C. (1993). Modernity, self-identity and the sequestration of death. Sociology, 27(3), 411–431. 10.1177/0038038593027003005

[r69] Meerwijk, E. L., & Weiss, S. J. (2014). Toward a unifying definition: Response to ‘the concept of mental pain’. Psychotherapy and Psychosomatics, 83(1), 62–63. 10.1159/000348869 24281693

[r70] Misra, N., & Srivastava, S. (2021). *The fallacy of happiness: A psychological investigation of suicide among successful people*. IntechOpen. 10.5772/intechopen.99425

[r71] Murray, S. A., Kendall, M., Grant, E., Boyd, K., Barclay, S., & Sheikh, A. (2007). Patterns of social, psychological, and spiritual decline toward the end of life in lung cancer and heart failure. Journal of Pain and Symptom Management, 34(4), 393–402. 10.1016/j.jpainsymman.2006.12.009 17616334

[r72] National Institute of Mental Health. (2025). *Suicide*. https://www.nimh.nih.gov/health/statistics/suicide

[r73] Neimeyer, R. A. (2005). Grief, loss, and the quest for meaning. Bereavement Care, 24(2), 27–30. 10.1080/02682620508657628

[r74] Neimeyer, R. A. (2016a). *Techniques in grief therapy: Assessment and intervention*. Routledge.

[r75] Neimeyer, R. A. (2016 b). Meaning reconstruction in the wake of loss: Evolution of a research program. Behaviour Change, 33(2), 65–79. 10.1017/bec.2016.4

[r76] Ng, Y. H., Jiao, K., Suen, M. H. P., Wang, J., & Chow, A. Y. M. (2025). The role of the social environment on dementia caregivers’ pre-death grief: A mixed-methods systematic review. Death Studies, 49(4), 359–378. 10.1080/07481187.2024.2329755 38497324

[r77] Pandey, B. R. (2025). Rethinking occupational health and safety principles — A systems perspective. Journal of the Royal Society of New Zealand, 55(6), 1362–1383. 10.1080/03036758.2024.2333555 40756835 PMC12315172

[r78] Patra, I., Muda, I., Ketut Acwin Dwijendra, N., Najm, M. A. A., Hamoud Alshahrani, S., Sajad Kadhim, S., Hameed, N. M., Alnassar, Y. S., Mohammed, N. M., Mustafa, Y. F., & Shojaeimotlagh, V. (2025). A systematic review and meta-analysis on death anxiety during COVID-19 pandemic. Omega, 91(3), 1079–1097. 10.1177/00302228221144791 37384902 PMC10311374

[r79] Pennington, M. (2001). *Memento mori. Eine Kulturgeschichte des Todes*. Kreuz.

[r80] Piaget, J. (1971). The theory of stages in cognitive development. In D. R. Green, M. P. Ford, & G. B. Flamer (Eds.). *Measurement and Piaget.* McGraw-Hill.

[r81] Ponterotto, J. G. (2015). Psychobiography in psychology: Past, present, and future. Journal of Psychology in Africa, 25(5), 379–389. 10.1080/14330237.2015.1101267

[r82] Ponterotto, J. G. (2025). *The psychobiographer’s handbook: A practical guide to research and ethics*. American Psychological Association.

[r83] Preti, A., & Miotto, P. (1999). Suicide among eminent artists. Psychological Reports, 84(1), 291–301. 10.2466/pr0.1999.84.1.291 10203964

[r84] Preti, A., De Biasi, F., & Miotto, P. (2001). Musical creativity and suicide. Psychological Reports, 89(3), 719–727. 10.2466/pr0.2001.89.3.719 11824743

[r85] Reynolds, J. D., Miller, S. P., & Maleh, N. T. (2023). Being and becoming: A psychobiography of James Baldwin. Journal of Personality, 91(1), 207–221. 10.1111/jopy.12743 35715894

[r86] Rolbiecki, A. J., Washington, K. T., Holman, J. G., & Lee, J. E. (2025). Sense making in the wake of familial death: “I continue to work through those feelings”. Death Studies, 49(1), 51–58. 10.1080/07481187.2023.2258509 37725580

[r87] Sabriseilabi, S. (2025). The color of death: An exploration of the effect of race and religion dimensions on attitudes toward euthanasia. Omega, 91(1), 455–473. 10.1177/00302228221135292 36264839

[r88] Sala, P. (2025). *Death and dying across the lifespan.* Paige Sala Misericordia University. https://digitalcommons.misericordia.edu/otd_capstone2025/17/

[r89] Schultz, W. T. (2005). *Handbook of psychobiography* (pp. 3–18). Oxford University Press.

[r90] Selman, L. (2024). Facing death differently: Revolutionising our approach to death and grief. BMJ (Clinical Research Ed.), 387, q2815. 10.1136/bmj.q2815 39715642

[r91] Shneidman, E. S. (1993). *Suicide as psychache: A clinical approach to self-destructive behavior*. Jason Aronson.

[r92] Shneidman, E. S. (1998). *The suicidal mind*. Oxford University Press.

[r93] Smith, M. F. (2014). “Suicidal mania” and flawed psychobiography: Two discussions of Virginia Woolf. English Studies, 95(5), 538–556. 10.1080/0013838X.2014.924279

[r94] Staples, J., & Widger, T. (2012). Situating suicide as an anthropological problem: Ethnographic approaches to understanding self-harm and self-inflicted death. Culture, Medicine and Psychiatry, 36, 183–203. 10.1007/s11013-012-9255-1 22395948

[r95] Stroebe, M., & Schut, H. (1999). The dual process model of coping with bereavement: Rationale and description. Death Studies, 23(3), 197–224. 10.1080/074811899201046 10848151

[r96] Stroebe, M., & Schut, H. (2010). The dual process model of coping with bereavement: A decade on. Omega, 61(4), 273–289. 10.2190/OM.61.4.b 21058610

[r97] Stroebe, M., & Schut, H. (2015). Family matters in bereavement: Toward an integrative intra-interpersonal coping model. Perspectives on Psychological Science: A Journal of the Association for Psychological Science, 10(6), 873–879. 10.1177/1745691615598517 26581741

[r99] Veatch, R. M., & Ross, L. F. (2016). *Defining death. The case for choice.* Georgetown University Press.

[r100] Visser, R. C. (2017). “Doing death”: Reflecting on the researcher’s subjectivity and emotions. Death Studies, 41(1), 6–13. 10.1080/07481187.2016.1257877 27845605

[r101] Walter, T. (1994). *The revival of death* (1^st^ ed.). Routledge. 10.4324/9780203220306

[r102] Weenolsen, P. (1991). Transcending the many deaths of life: Clinical implications for cure versus healing. Death Studies, 15(1), 59–80. 10.1080/07481189108252409

[r103] Weaver, J., & Wright, D. (2009). *Histories of suicide: International perspectives on self-destruction in the modern world.* University of Toronto Press.

[r104] Webb, R. T., Pickles, A. R., Appleby, L., Mortensen, P. B., & Abel, K. M. (2007). Death by unnatural causes during childhood and early adulthood in offspring of psychiatric inpatients. Archives of General Psychiatry, 64(3), 345–352. 10.1001/archpsyc.64.3.345 17339523

[r105] Wegner, B. R. (2020). *A psychobiography of Philip Brickman: The life, work, and human concerns of a social psychologist*. Chicago School of Professional Psychology. https://www.proquest.com/openview/c7bfe1b164c2597d7cc5fed5109f7dc1/1?pq-origsite=gscholar&cbl=18750&diss=y

[r106] World Health Organization. (2025). *Suicide*. https://www.who.int/news-room/fact-sheets/detail/suicide

[r107] Woodthorpe, K. (2009). Reflecting on death: The emotionality of the research encounter. Mortality, 14(1), 70–86. 10.1080/13576270802591228

[r108] Woodthorpe, K. (2011). Researching death: Methodological reflections on the management of critical distance. International Journal of Social Research Methodology, 14, 99–109. 10.1080/13645579.2010.496576

[r109] Yeh, A., & Trang, P. T. M. (2022). Avicii’s S.O.S.: A psychobiographical approach and corpus-based discourse analysis on suicidal ideation. Psychology of Language and Communication, 26(1), 207–241. 10.2478/plc-2022-0010

[r110] Young, M., & Cullen, L. (1996). *A good death: Conversations with East Londoners.* Routledge.

[r111] Zanalda, A. (1968). Psicobiografia di un suicidio [Psychobiography of a suicide (Virginia Woolf)]. Minerva Medica, 59(91), 4905–4909. 4882041

[r112] Zarei, F., Dehghan, M., & Shahrbabaki, P. M. (2025). The relationship between perception of good death with clinical competence of end-of-life care in critical care nurses. Omega, 91(1), 401–417. 10.1177/00302228221134721 36252601

